# Intracranial tumors of the central nervous system and air pollution – a nationwide case-control study from Denmark

**DOI:** 10.1186/s12940-020-00631-9

**Published:** 2020-07-08

**Authors:** Aslak Harbo Poulsen, Ulla Arthur Hvidtfeldt, Mette Sørensen, Robin Puett, Matthias Ketzel, Jørgen Brandt, Camilla Geels, Jesper H. Christensen, Ole Raaschou-Nielsen

**Affiliations:** 1 Danish Cancer Society Research Center, Strandboulevarden 49, DK-2100 Copenhagen Ø, Denmark; 2 Department of Natural Science and Environment, Roskilde University, Roskilde, Denmark; 3 Maryland Institute for Applied Environmental Health, University of Maryland School of Public Health, College Park, MD USA; 4 Department of Environmental Science, Aarhus University, Roskilde, Denmark; 5 Global Centre for Clean Air Research (GCARE) Department of Civil and Environmental Engineering, University of Surrey, Guildford, UK

**Keywords:** Epidemiology, Air pollution, Register study, CNS-tumors

## Abstract

**Background:**

Inconclusive evidence has suggested a possible link between air pollution and central nervous system (CNS) tumors. We investigated a range of air pollutants in relation to types of CNS tumors.

**Methods:**

We identified all (*n* = 21,057) intracranial tumors in brain, meninges and cranial nerves diagnosed in Denmark between 1989 and 2014 and matched controls on age, sex and year of birth. We established personal 10-year mean residential outdoor exposure to particulate matter < 2.5 μm (PM_2.5_), nitrous oxides (NO_X_), primary emitted black carbon (BC) and ozone. We used conditional logistic regression to calculate odds ratios (OR) linearly (per interquartile range (IQR)) and categorically. We accounted for personal income, employment, marital status, use of medication as well as socio-demographic conditions at area level.

**Results:**

Malignant tumors of the intracranial CNS was associated with BC (OR: 1.034, 95%CI: 1.005–1.065 per IQR. For NO_x_ the OR per IQR was 1.026 (95%CI: 0.998–1.056). For malignant non-glioma tumors of the brain we found associations with PM_2.5_ (OR: 1.267, 95%CI: 1.053–1.524 per IQR), BC (OR: 1.049, 95%CI: 0.996–1.106) and NO_x_ (OR: 1.051, 95% CI: 0.996–1.110).

**Conclusion:**

Our results suggest that air pollution is associated with malignant intracranial CNS tumors and malignant non-glioma of the brain. However, additional studies are needed.

## Background

Intracranial tumors of the central nervous system (CNS) are a heterogeneous group of tumors of primarily the meninges, the cranial nerves and the brain. Benign tumors are most often located in the meninges, and glioma is the most common malignant tumor type. The incidence of intracranial CNS tumor types differ by age, sex and race [1, 2]. Around 5% of these tumors are attributable to a range of hereditary syndromes [1]. While the only established exogenous risk factor is ionizing radiation, mounting evidence points toward a protective association with allergic and atopic conditions [1, 3, 4]. A range of medications has been investigated for potential associations [5–7] and some studies have suggested a link with exposures to pesticides or fertilizers [8–11], but the results are inconclusive. At present, no occupational or environmental risk factors for CNS tumors have been conclusively established [2, 12]. One candidate is, however, air pollution, which has been classified as “carcinogenic to humans” by the International Agency for Research on Cancer [13, 14] based primarily on mechanistic studies and epidemiological research demonstrating associations with lung cancer. Studies have indicated that ultrafine particles may reach the brain via the olfactory nerve or by crossing from the lungs to the blood stream and then passing the blood-brain barrier [15, 16]. Air pollution exposures have been associated with stroke and negative cognitive effects [17–20] and neuroimaging has shown links with reduced total brain and brain white matter volume [21].

Ambient air pollution consists of a complex array of substances that may be mutagenic/carcinogenic either directly or indirectly via induced inflammation and oxidative stress [16, 22]. As ultrafine particles (UFP) may reach the brain and due to their large number, small size and large surface area, scientific focus with regard to brain tumors has been on UFP and in a recent cohort study from Canada UFP exposure level was associated with risk of malignant brain tumors [23]. However, concentrations of airborne UFP are difficult to model. In Denmark, nitrogen oxides show good temporal correlation with UFP in street canyons [24, 25] and has,been used in epidemiological studies [26–28].

Several studies have investigated air pollution in relation to brain tumors with inconsistent results. An ecological study from the US found volatile organic compounds to be associated with county-level incidence of tumors of the CNS [29]. Two studies of a cohort from the US found no association between a range of air pollutants (particulate matter with a diameter less than 2.5 μm (PM2.5) and 10 μm (PM_10_), sulphur dioxide, nitrogen dioxide, carbon monoxide or ozone (O_3_)) and mortality from malignant tumors of the brain [30, 31]. In an exploratory analysis of 54,304 members of the Danish Diet Cancer and Health cohort, mean outdoor residential NO_x_ levels since 1971 were associated with an incidence rate ratio of 2.28 (95% confidence interval (CI): 1.25–4.19, cases = 95) per 100 μg/m^3^ increase, for tumors of the brain and central nervous system [26]. In a nationwide Danish study of 4183 cases diagnosed over the years 2000–2009, the odds ratio (OR) for a tumor (benign or malignant) of the brain was 1.11 (95% 0.84–1.4) per 100 μg/m^3^ increase in NO_x_ and the corresponding OR for non-glioma tumors of the brain was 1.53 (95% CI 1.02–2.29) [32]. The study indicated that increased brain tumor risk may only be detectable at high NO_x_ levels (> 80 μg/m^3^). The Danish Nurses Health Cohort study investigated intracranial CNS tumors (*n* = 121) and reported suggestions of a weak association with mean outdoor concentrations of particulate matter < 2.5 μm, PM < 10 μm, NO_2_ and NO_x_ over past 3 years. Restricting the analysis to meningioma or benign tumors increased risk estimates, but still with wide confidence intervals spanning the null [33]. The same tumor endpoints were investigated in the ESCAPE project that combined cohorts from six European countries, which found a suggestive evidence of an association between malignant tumors and long-term exposure to PM_2.5_ absorbance, a quantity closely related to elemental carbon and proposed by the authors to be a proxy for traffic related UFP [34].

In summary, some studies have found indications of an association between one or more air pollutants and one or more tumor types, however, the results are inconsistent. Possible explanations may relate to the relatively low number of cases but also to differences in tumor definitions, exposure assessment and the covariates adjusted for. We, therefore, conducted a large register-based case-control study of all intracranial CNS tumors, diagnosed in Denmark over the years 1989 to 2014 with an aim to investigate the different subtypes of tumors in relation to a range of air pollutants from a state-of-the-art exposure model.

## Materials and methods

The study was conducted in Denmark (population during the study period, approx. 5.4 million), were all citizens since 1968 have had a unique personal identification number, which allows individuals to be identified and tracked across all health and administrative registers in Denmark [35, 36].

### Case ascertainment

From the Danish Cancer Register, which holds nearly complete records of all cancer diagnoses in Denmark since 1943 [37, 38], we identified all Danes, aged 20 years or above, with a primary intracranial tumor (i.e. a tumor of the meninges, the cranial nerves, or the brain) in the period 1989 to 2014 Cases were not allowed to have other cancer diagnoses, except non-melanoma skin cancer, prior to their brain tumor.

### Sampling of controls

For each case we sampled two random controls, alive and without a cancer diagnosis at the date of diagnosis for their matched case (index-date), from the Danish Civil Registration System [39]. Controls were matched on sex and birth year and month.

### Exclusion criteria

Cases and controls were excluded if they had: 1) No recorded address in Denmark (excluding Greenland and the Faroe Islands) at index-date. 2) Less than 80% geocodable address history for the ten-year period preceding index-date. 3) Missing information on marital status, employment status, household disposable income or area of residence (parish). Information for some of these exclusions was available only after initial matching. Therefore, cases ending without controls or vice versa after full matching and exclusions were excluded.

### Exposure assessment

We extracted address histories for all cases and controls since 1979 from the Danish Civil Registration System [39] since air pollution modeling was possible from this year onwards. We geocoded all addresses, and front door air-pollutant concentrations at 2 m height were estimated for each of these addresses using the Danish DEHM/UBM/AirGIS modelling system [40, 41]. This integrated air pollution model system incorporates detailed time-varying information based on three air pollution contributions: 1) regional background, modelled with the DEHM model [42] on 150 km × 150 km scale covering the northern hemisphere and increasing resolution towards a 5.6 km × 5.6 km scale over Denmark, based on historical international and national emission data (including natural emissions); 2) urban background modelled with the UBM model [43] on 1 km × 1 km scale, based on high resolution emission data for Denmark for all emission sectors, land-use data and building heights; and 3) address-level local air pollution calculated with the OSPM model [44], based on street-level data on traffic type and intensity combined with emission factors and taking into account meteorology as well as street and building configuration. The model system is described in detail elsewhere [45, 46]. The model system provided yearly mean values of PM_2.5_, black carbon (BC – (a primary component of particulate matter), as well as the gases: O_3_, and NO_x_. The level of the latter correlates well with ultrafine particulate matter and has previously been utilized as a proxy for these [26–28] .

For each individual, we calculated a time-weighted average (TWA) concentration over residential addresses during the 10 years preceding the index-date.

### Covariates

From Statistics Denmark, we obtained yearly individual-level information on disposable income, marital status, employment status and country of origin. For all Danish parishes we obtained yearly information on percentage of the adult population: in lowest income quartile, unemployed, retired, doing manual labor, owning their own dwelling, living in social housing, being of Danish origin, previously convicted (theft, robbery, vandalism or violence), single parent families and having basic education as highest attained education level. In 1996, a total of 2160 parishes existed with a median number of 1032 inhabitants (range 33–35,979) and a mean area of 16.2 km^2^ (range 0.1–126.2). From the Danish National Prescription Register, which holds information on all prescription drugs redeemed in Denmark since 1995 [47], we obtained information on prescriptions for asthma or allergy conditions (ATC: R03, R06A) [47], prescriptions for hormone replacement therapy (HRT) (ATC: G03C, G03D, G03F, G03HB01), NSAIDs (ATC: B01AC06, B01AC30, N02BA01, N02BA51), non-aspirin NSAID (ATC: M01A except: M01AX), and antidiabetics (ATC: A10A, A10B). We identified all subjects who within a year redeemed at least two separate prescriptions for each drug to increase the likelihood that they were actually using the drug (NSAIDs and HRT) and/or that the indication was diabetes, asthma or allergy.

### Statistical methods

We used conditional logistic regression to calculate ORs for all intracranial CNS tumors (International Classification of Diseases, version 10 (ICD10): C70.0, C71.0-C71.9, C72.2, C72.5, D32.0,D33.0-D33.3,D42.0,D43.0-D43.3) and for the following subgroups: malignant tumors (prefix C), non-malignant tumors (prefix D), cranial nerves (C72.2,C72.5,D33.3,D43.3), meningioma (C70.0,D32.0,D42.0), glioma (morphology codes 9380/0–9480/9 within ICD10: C71.0-C71.9, D33.0-D33.3, D43.0-D43.2), malignant non-glioma brain tumors (all other morphologies within ICD10: C71.0-C71.9) and non-malignant non-glioma brain tumors (all other morphologies within ICD10: D33.0-D33.3, D43.0-D43.2). All pollutants were analyzed categorically as well as linearly since previous studies had suggested that effects may only be apparent among the highly exposed. In the categorical analyses, we used categories based on percentiles of exposure among controls: <50th percentile (reference), 50-94th percentile, 95-99th percentile and > 99th percentile. These categories provided a separate risk estimate for the few people with very high exposure, since a previous study found associations only in this group [32]. For all linear analyses, we tested for deviations from linearity by likelihood ratio testing comparing our model with a model including also the second-degree polynomial of the pollutant and with a model dividing the pollutant in 20 equal sized groups. We found no consistent signs of deviations (Supplement Table 1).

We analyzed data in three models: a crude model, only taking into account the matching factors; a model additionally adjusting for individual-level covariates, and a final, main model, which also included parish-level covariates. Individual-level covariates included personal income in deciles (calculated annually based on the distribution among controls), marital status (currently living together, formerly married or never married) and employment status (retired, unemployed, blue collar, low-level white collar, high-level white collar). The main, final model also included percentages of adult parish population in the lowest income quartile, unemployed, retired, doing manual labor, owning their own dwelling, living in social housing, being of Danish origin, previously convicted of a crime, single parent families and having basic education as highest attained education level. To minimize potential effects of prodromal symptoms, we assessed covariates 1 year before index-date.

We also conducted sensitivity analyses using the TWA exposure over 1 year and over 5 years. Finally, in an analysis restricting to cases diagnosed after 1995, we adjusted for ever-use of HRT [1, 6] NSAID and non-aspirin NSAID [5], which some studies have associated with CNS tumors and Danish administrative data suggest higher usage in more urbanized regions. We also adjusted for use of antidiabetics, and medication for asthma or allergy conditions since studies have suggested that both diabetes and allergy could be associated with a decreased risk for types of CNS tumors and some studies also associate these conditions with air pollution [48, 49].

Statistical analysis was performed in SAS 9.3 (SAS Institute Inc., Cary, NC, USA).

## Results

We identified 22,454 adult cases diagnosed with a primary intracranial CNS tumor in the period 1989 to 2014 and 44,908 matched controls. We excluded cases (45 / 0.2%) and controls (3567 / 7.9%) not living in Denmark at the time of diagnosis, as well as cases (865 / 3.9%) and controls (2318 / 5.2%) who had less than 80% geocodable address history in Denmark for the period 10 years before index-date. We also excluded 42 cases (0.2%) and 126 controls (0.3%) due to missing data on one or more covariates. Finally, we excluded 445 cases (2.0%) with no remaining controls and 1529 controls (3.4%) with no case, after the above exclusions. The resulting population comprised 21,057 cases and 37,368 controls. Of these cases, 7465 were glioma and 5657 were meningioma. Malignant tumors comprised 46% of the cases.

Table 1 shows virtually no differences between cases and controls for any covariate. Similarly, Table 2 shows similar distributions of the air pollutants for cases and controls below the 99th percentile. However, the maximum observed concentrations of BC, PM_2.5_ NO_2_ and NO_x_ were higher among cases. The correlation coefficients between air pollutants were in the range between − 0.98 and 0.94 (Table 3).

**Table 1:** Descriptive characteristics of intracranial CNS tumor cases and matched controls. Denmark, 1989–2014

	CASES	CONTROLS
**TOTAL**	21,057	37,368
**Individual level factors:**		
**FEMALE**	53%	54%
**Age at index date**^**a**^		
median	62	63
10th pctl	39	39
90th pctl	80	80
**Region of origin**		0
Denmark	96%	95%
Non-Western	2%	2%
Western	3%	3%
**Marital status**		
Living together	61%	59%
Previously married	25%	26%
Never married	14%	14%
**Occupational status**		
Unemployed	4%	4%
Low skill level	23%	22%
Medium skill level	16%	16%
High skill level	8%	8%
Retired	49%	50%
**Disposable income (dKK)**		
Median	125,757	122,812
10th pctl	61,311	58,995
90th pctl	254,411	254,053
**At least 2 prescriptions within a year (data only available since 1996)**		
Non-aspirin NSAID	28%	27%
Aspirin	12%	13%
Antidiabetics	4%	4%
HRT	13%	12%
Allergy medication	8%	8%
**Parish level factors:**		
**% of population with only basic education**		
Median	27	27
10th pctl	15	15
90th pctl	41	42
**% of population in manual labor**		
Median	29	29
10th pctl	18	18
90th pctl	38	38
**% of population retired**		
Median	6	6
10th pctl	3	3
90th pctl	11	11
**% of population unemployed**		
median	4	4
10th pctl	1	1
90th pctl	8	8
**% of population in 1st income quartile**		
Median	10	10
10th pctl	5	5
90th pctl	18	18
**% of population in social housing**		
Median	13	13
10th pctl	0	0
90th pctl	43	43
**% of population owning own dwelling**		
Median	65	66
10th pctl	29	30
90th pctl	92	92
**% single parent families**		
Median	5	5
10th pctl	3	3
90th pctl	7	7
**% of population of Danish origin**		
Median	94	95
10th pctl	84	85
90th pctl	98	98
**% of population previously convicted**		
Median	0	0
10th pctl	0	0
90th pctl	1	1

a: index date = date of diagnosis of matched case

**Table 2:** Descriptive data on air pollutants among intracranial CNS tumor cases and controls in Denmark, 1989–2014

10 year time-weighted mean exposure	min	1st pctl	5th pctl	10th pctl	25th pctl	median	75th pctl	90th pctl	95th pctl	99th pctl	max	Inter quartile range
**BC (μg/m**^**3**^**)**												
Controls	0.29	0.37	0.43	0.47	0.55	**0.72**	0.94	1.21	1.49	2.42	8.00	0.39
Cases	0.29	0.38	0.43	0.47	0.56	**0.73**	0.94	1.21	1.50	2.43	13.64	0.38
**PM**_**2.5**_**(μg/m**^**3**^**)**												
Controls	9.91	11.83	12.81	13.41	14.67	**16.87**	20.07	22.45	23.53	26.30	37.09	5.39
Cases	9.93	11.82	12.83	13.40	14.63	**16.76**	19.95	22.43	23.52	26.06	41.46	5.31
**NO**_**2**_**(μg/m**^**3**^**)**												
Controls	5.68	8.12	10.35	11.79	14.57	**25.35**	19.18	31.06	35.72	48.33	73.86	10.78
Cases	5.66	8.20	10.39	11.85	14.79	**25.60**	19.35	31.22	35.74	48.26	79.00	10.81
**NO**_**x**_**(μg/m**^**3**^**)**												
Controls	6.13	8.84	11.42	13.17	17.06	**24.83**	35.92	51.01	67.33	116.69	270.31	18.86
Cases	6.15	8.93	11.48	13.26	17.34	**25.15**	36.35	51.35	67.22	115.37	330.91	19.01
**O**_**3**_**(μg/m**^**3**^**)**												
Controls	15.82	37.5	47.04	50.70	55.58	**60.92**	65.30	68.44	70.26	73.81	77.45	9.72
Cases	15.72	37.05	47.08	50.63	55.43	**60.78**	65.12	68.38	70.33	73.56	77.68	9.69

**Table 3:** Pearson correlations between pollutants

	10 year mean BC (μg/m^3^)	10 year mean PM_**2.5**_ (μg/m^3^)	10 year mean NO_**X**_ (μg/m^3^)	10 year mean NO_**2**_ (μg/m^3^)	10 year mean O_**3**_ (μg/m^3^)
**Air pollutants**					
10 year mean O_3_ (μg/m^3^)	−0.85	−0.62	−0.88	−0.98	1.00
10 year mean NO_2_ (μg/m^3^)	0.88	0.57	0.92	1.00	
10 year mean NO_X_ (μg/m^3^)	0.94	0.55	1.00		
10 year mean PM_2.5_ (μg/m^3^)	0.54	1.00			
10 year mean BC (μg/m^3^)	1.00				

In linear analysis, the ORs for association with total intracranial CNS tumors was 0.967 (95%CI: 0.934–1.002) for O_3_, 1.011 (95%CI: 0.992–1.030) for NO_x_ 1.031 (95%ci: 0.997–1.066) for NO_2_ and 1.016 (95%CI: 0.996–1.037) for BC. For BC, the categorical results showed an exposure response pattern.

Malignant tumors of the intracranial CNS was associated with BC (OR: 1.034, 95%CI: 1.005–1.065 per IQR), NO_2_ (OR: 1.042, 95%CI: 0.992–1.095 per IQR) and NO_x_ (OR: 1.026, 95%CI: 0.998–1.056 per IQR). Except for NO_2_, the highest risk estimates were observed for the 95-99th percentile of exposures in the categorical analysis (Table 4).

**Table 4:** Fully adjusted, categorical and linear associations between time-weighted average air pollution (10 years before index date) and risk of intracranial CNS tumors, Denmark 1989–2014

Exposure levels (a)	NO_**X**_			NO_**2**_			BC			PM_**2.5**_			O_**3**_		
	Cntrls	Cases	OR (95%CI)	Cntrls	Cases	OR	Cntrls	Cases	OR (95%CI)	Cntrls	Cases	OR (95%CI)	Cntrls	Cases	OR (95%CI)
**Intracranial CNS tumors**															
< median	18,681	10,341	Referent	18,674	10,367		18,818	10,386	Referent	18,682	10,745	Referent	18,690	10,694	Referent
50-94pct	16,815	9665	1.017 (0.973–1.063)	16,823	9631	1.004 (0.959–1.051)	16,692	9605	1.021 (0.977–1.068)	16,816	9263	0.991 (0.926–1.062)	16,806	9288	0.986 (0.941–1.033)
95-99pct	1496	845	1.021 (0.927–1.126)	1495	847	1.001 (0.904–1.109)	1481	850	1.045 (0.948–1.152)	1494	865	1.069 (0.948–1.205)	1496	885	1.059 (0.960–1.168)
≥ 99pct	373	204	0.993 (0.828–1.191)	373	210	1.006 (0.838–1.209)	374	214	1.057 (0.884–1.265)	373	182	0.897 (0.736–1.094)	373	188	0.902 (0.748–1.088)
per IQR			1.011 (0.992–1.030)			1.031 (0.997–1.066)			1.016 (0.996–1.037)			1.010 (0.944–1.08)			0.967 (0.934–1.002)
**Malignant**															
< median	8630	4828	Referent	8658	4877		8776	4887	Referent	8121	4665	Referent	8801	4945	Referent
50-94pct	7923	4451	1.006 (0.943–1.074)	7882	4391	0.982 (0.919–1.051)	7772	4373	1.022 (0.957–1.091)	8335	4560	1.014 (0.917–1.122)	7752	4328	1.006 (0.939–1.077)
95-99pct	677	416	1.109 (0.961–1.279)	691	415	1.047 (0.902–1.216)	681	424	1.155 (1.003–1.331)	766	470	1.192 (1.008–1.411)	685	414	1.082 (0.937–1.249)
≥ 99pct	167	90	0.972 (0.740–1.275)	166	102	1.070 (0.819–1.399)	168	101	1.108 (0.853–1.441)	175	90	0.981 (0.737–1.305)	159	98	1.072 (0.818–1.404)
per IQR			1.026 (0.998–1.056)			1.042 (0.992–1.095)			1.034 (1.005–1.065)			1.021 (0.926–1.126)			0.962 (0.913–1.012)
**Non-malignant**															
< median	10,051	5513	Referent	10,016	5490		10,042	5499	Referent	10,561	6080	Referent	9889	5749	Referent
50-94pct	8892	5214	1.027 (0.967–1.091)	8941	5240	1.023 (0.961–1.089)	8920	5232	1.022 (0.961–1.086)	8481	4703	0.971 (0.883–1.067)	9054	4960	0.968 (0.908–1.031)
95-99pct	819	429	0.953 (0.834–1.090)	804	432	0.965 (0.838–1.111)	800	426	0.960 (0.839–1.099)	728	395	0.949 (0.799–1.127)	811	471	1.039 (0.909–1.188)
≥ 99pct	206	114	1.013 (0.793–1.294)	207	108	0.961 (0.747–1.237)	206	113	1.011 (0.791–1.292)	198	92	0.834 (0.633–1.100)	214	90	0.771 (0.593–1.002)
per IQR			0.998 (0.972–1.025)			1.022 (0.976–1.070)			1.000 (0.972–1.028)			1.000 (0.912–1.096)			0.972 (0.926–1.021)
**Glioma**															
< median	6596	3713	Referent	6606	3741		6684	3754	Referent	6309	3703	Referent	6512	3734	Referent
50-94pct	5899	3406	1.025 (0.951–1.105)	5876	3369	1.003(0.929–1.083)	5816	3352	1.036 (0.961–1.117)	6101	3379	0.929 (0.827–1.043)	5954	3338	0.991 (0.916–1.071)
95-99pct	478	284	1.064 (0.897–1.262)	491	288	1.013(0.848–1.209)	477	294	1.129 (0.954–1.336)	561	327	0.994 (0.816–1.210)	501	313	1.101 (0.932–1.299)
≥ 99pct	118	62	0.925 (0.668–1.281)	118	67	0.962 (0.697–1.328)	114	65	1.022 (0.741–1.410)	120	56	0.773 (0.545–1.097)	124	80	1.100 (0.812–1.490)
per IQR			1.017 (0.983–1.052)			1.026 (0.969–1.087)			1.028 (0.993–1.063)			0.938 (0.836–1.053)			0.973 (0.916–1.033)
**Meningioma**															
< median	5164	2667	Referent	5132	2608		5163	2627	Referent	5446	3099	Referent	4966	3010	Referent
50-94pct	4409	2701	1.087 (0.869–1.122)	4450	2757	1.133 (1.036–1.239)	4429	2748	1.123 (1.030–1.224)	4210	2326	0.943 (0.827–1.075)	4569	2376	0.920 (0.841–1.007)
95-99pct	412	234	1.045 (0.604–1.149)	410	239	1.100 (0.906–1.336)	394	225	1.085 (0.898–1.310)	340	195	0.992 (0.779–1.264)	445	226	0.922 (0.763–1.115)
≥ 99pct	109	55	0.934 (0.650–2.356)	102	53	1.004 (0.699–1.440)	108	57	1.036 (0.736–1.460)	98	37	0.651 (0.428–0.990)	114	45	0.748 (0.519–1.078)
per IQR			1.009 (0.972–1.047)			1.083 (1.016–1.154)			1.016 (0.977–1.057)			1.088 (0.955–1.239)			0.923 (0.862–0.988)
**Cranial Nerves**															
< median	2181	1280	Referent	2167	1276		2147	1286	Referent	2309	1335	Referent	2121	1192	Referent
50-94pct	1943	1104	0.987 (0.869–1.122)	1960	1108	0.978 (0.856–1.119)	1974	1096	0.915 (0.804–1.042)	1823	1045	1.047 (0.854–1.283)	1989	1156	0.990 (0.864–1.133)
95-99pct	159	69	0.833 (0.604–1.149)	151	69	0.870 (0.622–1.217)	161	75	0.814 (0.596–1.111)	146	74	0.912 (0.614–1.353)	160	102	1.131 (0.840–1.522)
≥ 99pct	28	17	1.237 (0.650–2.356)	33	17	1.052 (0.559–1.978)	29	13	0.816 (0.408–1.635)	33	16	0.971 (0.501–1.884)	41	20	0.849 (0.476–1.517)
per IQR			0.970 (0.908–1.036)			0.952 (0.858–1.058)			0.943 (0.879–1.011)			0.963 (0.776–1.194)			1.046 (0.936–1.169)
**Malignant Non-glioma tumors of the brain**															
< median	2031	1121	Referent	2040	1142		2085	1138	Referent	1851	1001	Referent	2235	1184	Referent
50-94pct	1977	1021	1.021 (0.977–1.068)	1969	1000	0.904 (0.786–1.041)	1913	1000	0.966 (0.842–1.108)	2156	1128	1.254 (1.019–1.543)	1793	995	1.059 (0.918–1.221)
95-99pct	189	131	1.045 (0.948–1.152)	191	125	1.153 (0.867–1.534)	197	125	1.219 (0.933–1.593)	191	139	2.033 (1.451–2.849)	184	102	1.003 (0.750–1.342)
> =99pct	50	28	1.057 (0.884–1.265)	47	34	1.267 (0.774–2.075)	52	38	1.374 (0.867–2.177)	49	33	1.833 (1.093–3.073)	35	20	1.054 (0.587–1.893)
per IQR			1.049 (0.996–1.106)			1.084 (0.983–1.194)			1.051 (0.996–1.110)			1.267 (1.053–1.524)			0.937 (0.846–1.037)
**Non-malignant Non-glioma tumors of the brain**															
< median	2709	1560	Referent	2729	1600		2739	1581	Referent	2767	1607	Referent	2856	1574	Referent
50-94pct	2587	1433	0.958 (0.854–1.075)	2568	1397	0.894(0.793–1.008)	2560	1409	0.944 (0.840–1.060)	2526	1385	0.997 (0.828–1.201)	2501	1423	1.033 (0.915–1.167)
95-99pct	258	127	0.875 (0.685–1.118)	252	126	0.803 (0.618–1.043)	252	131	0.909 (0.712–1.161)	256	130	0.895 (0.655–1.222)	206	142	1.282 (0.995–1.652)
≥ 99pct	68	42	1.074 (0.707–1.633)	73	39	0.864 (0.562–1.329)	71	41	1.000 (0.659–1.518)	73	40	1.002 (0.637–1.574)	59	23	0.743 (0.442–1.250)
per IQR			0.995 (0.950–1.042)			0.969(0.890–1.054)			1.003 (0.956–1.053)			0.897 (0.759–1.060)			1.012 (0.925–1.107)

a: interquartile range (IQR) and percentiles calculated among all controls. For specific values, see Table 2

b: Matched on age, sex and month of birth and adjusted for marital status, occupational status, personal income, region of origin and area level information on % of parish population with income in lowest quartile, unemployed, manual labor, retired, basic education, living in social housing, owning their own dwelling, single parent families, previously convicted, of Danish origin

Meningioma was inversely associated with O_3_ (OR: 0.923, 95%CI: 0.862–0.988), which was also indicated by the categorical analysis. For NO2 there was a positive linear association (OR: 1.083, 95%CI: 1.016–1.154) this was not apparent in categorical analysis. For the other pollutants, there was no evidence of association in linear analysis. Neither was there evidence of associations in categorical analysis, except for the highest percentile of PM_2.5_ exposure where the OR was 0.65 (95%CI: 0.43–0.99). In linear analysis, the corresponding OR was 1.088 (95%CI: 0.955–1.239) (Table 4).

For malignant non-glioma tumors of the brain we found an association with PM_2.5_ (OR: 1.267, 95%CI: 1.053–1.524 per IQR), and all PM_2.5_ exposure categories above the median were associated with elevated risks with some suggestion of exposure-response. For BC, NO2 and NO_x_ the corresponding ORs in the linear analyses were 1.049 (95%CI: 0.996–1.106), 1.267(95%CI: 0.983–1.194) and 1.051 (95% CI: 0.996–1.110), respectively (Table 4).

There was no consistent evidence of any associations in either the linear or the categorical analyses for subgroups not mentioned above (Table4).

Adjusting for area-level covariates generally attenuated risk estimates (Table 5). This was particularly notable for meningioma, where all pollutants were significantly associated in the crude and individual level adjusted models but only the associations with NO_2_ and O_3_ remained statistically significant after additional adjustment for parish level covariates. An exception from this pattern was malignant non-glioma tumors of the brain, for which the risk estimates increased both when including individual and area level factors in the model.

**Table 5:** Effects of confounder adjustment on linear associations between time-weighted average air pollution (10 years before index date) and risk of intracranial CNS tumors, Denmark 1989–2014

Air pollutant	IQR (μg/m3)	Model 1^**a**^		Model 2^**b**^		Model 3^**c**^	
		OR pr IQR	95%CI	OR pr IQR	95%CI	OR pr IQR	95%CI
**Intracranial CNS tumors***(Cases: 21055 Controls: 37356)*							
NO_x_	18.86	1.020	(1.004–1.036)	1.025	(1.009–1.041)	1.011	(0.992–1.030)
NO_2_	10.78	1.041	(1.017–1.066)	1.052	(1.027–1.077)	1.031	(0.997–1.066)
BC	0.39	1.025	(1.008–1.042)	1.030	(1.013–1.048)	1.016	(0.996–1.037)
PM_2.5_	5.39	1.042	(0.981–1.107)	1.053	(0.991–1.119)	1.010	(0.944–1.080)
O_3_	9.72	0.957	(0.934–0.981)	0.948	(0.924–0.972)	0.967	(0.934–1.002)
**Malignant***(Cases: 9785 Controls: 17397)*							
NO_x_	18.86	1.025	(1.001–1.049)	1.035	(1.011–1.060)	1.026	(0.998–1.056)
NO_2_	10.78	1.033	(0.999–1.069)	1.051	(1.015–1.089)	1.042	(0.992–1.095)
BC	0.39	1.030	(1.006–1.056)	1.040	(1.015–1.066)	1.034	(1.005–1.065)
PM_2.5_	5.39	1.022	(0.936–1.116)	1.041	(0.953–1.138)	1.021	(0.926–1.126)
O_3_	9.72	0.968	(0.934–1.003)	0.952	(0.918–0.987)	0.962	(0.913–1.012)
**Non-malignant***(Cases: 11270 Controls: 19968)*							
NO_x_	18.86	1.015	(0.993–1.037)	1.017	(0.995–1.039)	0.998	(0.972–1.025)
NO_2_	10.78	1.048	(1.015–1.982)	1.054	(1.020–1.089)	1.022	(0.976–1.070)
BC	0.39	1.020	(0.997–1.044)	1.022	(0.998–1.046)	1.000	(0.972–1.028)
PM_2.5_	5.39	1.061	(0.977–1.153)	1.065	(0.980–1.158)	1.000	(0.912–1.096)
O_3_	9.72	0.948	(0.917–0.980)	0.943	(0.911–0.975)	0.972	(0.926–1.021)
**Glioma***(Cases: 7465 Controls: 13091)*							
NO_x_	18.86	1.021	(0.994–1.050)	1.034	(1.005–1.063)	1.017	(0.983–1.052)
NO_2_	10.78	1.031	(0.990–1.073)	1.052	(1.010–1.096)	1.026	(0.969–1.087)
BC	0.39	1.029	(1.000–1.059)	1.041	(1.011–1.071)	1.028	(0.993–1.063)
PM_2.5_	5.39	0.964	(0.868–1.069)	0.984	(0.886–1.093)	0.938	(0.836–1.053)
O_3_	9.72	0.968	(0.929–1.009)	0.949	(0.909–0.990)	0.973	(0.916–1.033)
**Meningioma***(Cases: 5657 Controls: 10094)*							
NO_x_	18.86	1.046	(1.014–1.078)	1.044	(1.012–1.077)	1.009	(0.972–1.047)
NO_2_	10.78	1.133	(1.083–1.185)	1.132	(1.082–1.185)	1.083	(1.016–1.154)
BC	0.39	1.058	(1.024–1.093)	1.056	(1.022–1.092)	1.016	(0.977–1.057)
PM_2.5_	5.39	1.211	(1.076–1.363)	1.202	(1.067–1.354)	1.088	(0.955–1.239)
O_3_	9.72	0.878	(0.837–0.920)	0.878	(0.836–0.921)	0.923	(0.862–0.988)
**Cranial Nerves***(Cases: 2470 Controls: 4311)*							
NO_x_	18.86	0.953	(0.903–1.005)	0.960	(0.909–1.014)	0.970	(0.908–1.036)
NO_2_	10.78	0.940	(0.873–1.011)	0.945	(0.877–1.019)	0.952	(0.858–1.058)
BC	0.39	0.941	(0.889–0.996)	0.946	(0.893–1.002)	0.943	(0.879–1.011)
PM_2.5_	5.39	0.966	(0.799–1.168)	0.969	(0.799–1.175)	0.963	(0.776–1.194)
O_3_	9.72	1.057	(0.979–1.141)	1.054	(0.975–1.141)	1.046	(0.936–1.169)
**Malignant Non-glioma tumors of brain proper***(Cases: 2301 Controls: 4247)*							
NO_x_	18.86	1.034	(0.991–1.080)	1.038	(0.994–1.084)	1.049	(0.996–1.106)
NO_2_	10.78	1.036	(0.969–1.107)	1.047	(0.978–1.120)	1.084	(0.983–1.194)
BC	0.39	1.035	(0.989–1.082)	1.038	(0.992–1.086)	1.051	(0.996–1.110)
PM_2.5_	5.39	1.179	(0.998–1.393)	1.191	(1.007–1.409)	1.267	(1.053–1.524)
O_3_	9.72	0.975	(0.910–1.045)	0.965	(0.899–1.035)	0.937	(0.846–1.037)
**Non-malignant Non-glioma tumors of brain proper***(Cases: 3162 Controls: 5622)*							
NO_x_	18.86	1.001	(0.964–1.040)	1.007	(0.969–1.047)	0.995	(0.950–1.042)
NO_2_	10.78	0.990	(0.933–1.049)	1.005	(0.947–1.067)	0.969	(0.890–1.054)
BC	0.39	1.006	(0.996–1.047)	1.013	(0.972–1.055)	1.003	(0.956–1.053)
PM_2.5_	5.39	0.920	(0.792–1.068)	0.947	(0.815–1.101)	0.897	(0.759–1.060)
O_3_	9.72	1.000	(0.941–1.064)	0.982	(0.923–1.046)	0.994	(0.920–1.073)

^a^Crude model, adjusted for age, sex and month of birth by matching

^b^Model 1 with additional adjustment individual level data on marital status, occupational status, personal income, region of origin

^c^Model 2 with additional adjustment for area level information on % of parish population with income in lowest quartile, unemployed, manual labor, retired, basic education, living in social housing, owning their own dwelling, single parent families, previously convicted, of Danish origin

We also analyzed air pollutant exposure averaged over 1 year and 5 years prior to diagnosis (Supplement Table 2). For malignant tumors, there were indications that the risk estimates were lower in association with shorter averaging periods.

Adjusting for use of NSAIDs, HRT, antidiabetic medication and allergy medication left ORs virtually unchanged in a sensitivity analysis of cases diagnosed after 1995 (Supplement Table 3).

## Discussion

In this nationwide study, the largest to date, with more than 20,000 intracranial CNS tumor cases, we found PM_2.5_ air pollution NO_x_ and BC to be associated with malignant non-glioma tumors of the brain. BC and NO_2_ were weakly associated with increased risk for malignant intracranial CNS tumors and O_3_ was inversely associated with risk for meningioma. Total intracranial CNS tumors were associated with BC air pollution in an exposure-response manner. ORs for benign tumors were sensitive to adjustment.

Some previous studies on air pollutants and tumors of the brain or CNS found positive associations, whereas others did not. When accounting for the difference of scale, the results have confidence intervals that overlap, indicating that they are compatible. In general, this is also the case when comparing the present study with previous studies (For the reader’s convenience, results from our previous studies, rescaled to the IQR of the present study, can be found as supplement Table 4).

In a Danish cohort, cancer of the brain was associated with NO_x_ (rescaled to IQR of the present study: HR 1.17, 95% CI: 1.04–1.31 per 18.88 μg/m^3^) [26]. The present study could not confirm such an association for the wider group of intracranial CNS tumors. The present study, however, suggested that NO_2_ and BC may be associated with a small increased risk for malignant intracranial CNS tumors. No other study has investigated BC in relation to intracranial CNS tumors. However, in the multinational ESCAPE study, the authors found suggestive evidence that PM_2.5_ absorbance, a proxy similar to BC, was associated with malignant CNS tumors, although with a wide confidence interval spanning the null [34]. For NO_2_ the HR in that study were near identical to the ORs of the present study. A recent Canadian study found no evidence of an association between PM_2.5_ or NO_2_ and malignant brain tumors [23]. Turner et al. [30] reported on malignant tumors of the brain, adjusted for a comprehensive array of personal and area-level confounders, and found no significant association with PM_2.5_, NO_2_ or O_3_. When accounting for the different unit scales, the HRs in that study were very similar to those observed in our study.

In a Danish register-based investigation of tumors of the brain, non-glioma tumors were associated with NO_x_ (rescaled to the IQR of the present study: OR 1.08, 95%CI: 1.004–1.169 per 18.88 μg/m^3^) [32]. The cases of that study were also part of the present study, and we found risk estimates of similar magnitude although not statistically significant for NO_x_ and BC. For PM_2.5_ we found a stronger association.

We found inverse associations between ozone and meningioma. We cannot provide a plausible explanation for a causal inverse association and it could reflect the inverse relationship between O_3_ and other pollutants, although chance is also a possible explanation. Two previous studies, conducted on subsets of the cases in the present study, have found some indication of associations between other air pollutants and benign brain tumors. Small sample size and lack of confounder adjustment (in one study) mean that these observations may result from chance or confounding [32, 33].

Use of the comprehensive and virtually complete registers on the Danish population [35, 36] was a major strength of our study and allowed us to include all cases in Denmark over a 26-year period. In addition, we were able to investigate subgroups of CNS tumors and to identify even weak associations. The Danish registers provided nearly complete address histories for the vast majority of participants. These address histories in combination with a state-of-the-art air pollution model system allowed us to estimate TWA concentration of outdoor pollution at addresses over the ten years prior to diagnosis, but we had no information about occupational exposures. However, previous studies found little or no influence on risk estimates from adjustment for occupation in the petrochemical industry [26, 34]. We estimated exposure with a state-of-the-art integrated air pollution system, which has shown good prediction of both temporal and spatial variation down to the street scale [46, 50, 51]. Even so, some non-differential exposure misclassification is inevitable and may have driven risk estimates towards the null. It is possible that our 10-year TWA metrics may not optimally capture the relevant time window of air pollution exposure. However, for malignant tumors and for malignant non-glioma tumors of the brain, shorter TWA-periods were associated with lower risk estimates, suggesting that an association is better captured by the 10 year TWA-period. It was a major strength of our study that we in a nationwide study, could adjust in detail for personal socioeconomic position, medication use and neighborhood socio-demographic factors. However, we cannot rule out that confounding from unknown risk factors or chance may have affected our findings, particularly since we performed a large number of analyses. It was a potential limitation that we did not have information on BMI, and smoking, which some studies associated with glioma or meningioma. We also lacked information on ionizing radiation, which is an established risk factor for CNS tumors. We, however, consider the potential bias small, as we can think of no obvious route of association with air pollution in a Danish context, beyond what is addressed by socioeconomic covariates. A potential limitation for the benign tumors is that we had no information on scanning procedures prior to diagnosis. It may have affected our results for benign tumors if the quality or likelihood of scanning differed by air pollution level; some benign tumors can be symptom free for many years and may only be detected by chance during routine scanning [52]. We did not account for pre-existing genetic conditions associated with risk for CNS tumors. If families with such genetic syndromes are more likely to live in urban or rural areas, it may have affected risk estimates. However, the size of this potential bias is limited by the low population prevalence of such conditions and the small proportion of CNS tumors related to such syndromes.

The case-populations of the three previous Danish studies [26, 32, 33], as well as parts of the multinational ESCAPE study [34] are nested within the population of the present study. Similar errors or random effects may therefore have influenced the results of these studies, which constitute the majority of publications on residential air-pollution and CNS-tumors. In our crude model, ORs were elevated for both benign and malignant tumors. Adjustment for area-level SES brought ORs for benign tumors close to unity whereas the ORs for malignant tumors remained elevated. Benign and malignant tumors are generally different diseases and may have different etiologies. Some benign tumors may go undetected for years or even for life. Our results indicate that the likelihood of receiving brain scans, which could discover benign tumors, is associated with some area level socioeconomic factor that is also associated with higher levels of air pollution. Our results for malignant tumors are more likely to reflect true association, as these tumors will typically be detected due to symptoms and not as chance findings at brain scans.

On the whole, our results suggest that if any association exists between air pollution and CNS tumors, it is likely weak. For benign tumors, adequate confounder control appears to be particularly important.

## Conclusion

The present study is the largest study to date on air pollutants and intracranial tumors of the CNS, and indicates that air pollution is a risk factor for CNS tumors. Our data suggest that, the most likely relationship is with malignant intracranial CNS tumors and malignant non-glioma tumors of the brain. However, additional independent studies are needed.

## Supplementary information

**Additional file 1: Table 1.** Tests for linearity. *P*-values for likelihood ratio tests comparing fully adjusted model linear model with model including also a quadratic term of the pollutant (A) and a categorical model with twenty equal sized categories of exposure (defined among controls) (B). Air pollution averaged over all addresses 10 years prior to index. **Table 2.** Linear associations between time-weighted average air pollution over 1, 5 and 10-year periods and risk of intracranial CNS tumors, Denmark 1989–2014 **Table 3.** Linear associations between mean air pollution (10 years before diagnosis) and incidence of intracranial CNS tumors with and without adjustment for drug use, Denmark 1996–2014 **Table 4.** Summaries of results from our previous studies rescaled to the IQRs of the present study: PM_2.5_ = 5.39 μg/m^3^, NO_x_ = 18.86 μg/m^3^, NO_2_ = 10.78 μg/m^3^.

## Data Availability

Data is located at the secure IT environment at Statistics Denmark. Access to data at Statistics Denmark can be obtained if approved by Statics Denmark. Requirements include compliance with the EU General Data Protection Regulation and the Danish Data Protection Act.

## Abbreviations

ATC: Anatomical therapeutic chemical classification system,
BC: Primary emitted black carbon
CI: Confidence interval
CNS: Central nervous system
HRT: Hormone replacement therapy
ICD: International classification of diseases
IQR: Interquartile range
NO_x_: Nitrous oxides
NO_2_: Nitrogen dioxide
NSAID: Non steroid anti-inflammatory drug
O_3_: Ozone
OR: Odds ratio
PM_10_: PM < 10 μm
PM_2.5_: Particulate matter < 2.5 μm
TWA: Time-weighted average
UFP: Ultrafine particles
