# Supplementary material for: Intracranial tumors of the central nervous system and air pollution – a nationwide case-control study from Denmark
Source: Environ Health. 2020 Jul 8;19:81. doi: 10.1186/s12940-020-00631-9 (PMC7346389; doi:10.1186/s12940-020-00631-9)
Supplement: Supplementary file 1 — Additional file 1: Table 1. Tests for linearity. P-values for likelihood ratio tests comparing fully adjusted model linear model with model including also a quadratic term of the pollutant (A) and a categorical model with twenty equal sized categories of exposure (defined among controls) (B). Air pollution averaged over all addresses 10 years prior to index. Table 2. Linear associations between time-weighted average air pollution over 1, 5 and 10-year periods and risk of intracranial CNS tumors, Denmark 1989–2014 Table 3. Linear associations between mean air pollution (10 years before diagnosis) and incidence of intracranial CNS tumors with and without adjustment for drug use, Denmark 1996–2014 Table 4. Summaries of results from our previous studies rescaled to the IQRs of the present study: PM2.5 = 5.39 μg/m3, NOx = 18.86 μg/m3, NO2 = 10.78 μg/m3. [file 12940_2020_631_MOESM1_ESM.docx]

**Supplement table 1**

Tests for linearity. P-values for likelihood ratio tests comparing fully adjusted model linear model with model including also a quadratic term of the pollutant (A) and a categorical model with twenty equal sized categories of exposure (defined among controls) (B). Air pollution averaged over all addresses 10 years prior to index.

|  | **BC** | **PM_2.5_** | **NO_2_** | **NO_x_** | **O_3_** |
| --- | --- | --- | --- | --- | --- |
| ***A: Including 2nd degree polynomial*** | | | | | |
| Intracranial CNS tumors | 0.40 | 0.16 | 0.55 | 0.33 | 0.32 |
| Malignant | 0.05 | 0.48 | 0.46 | 0.53 | 0.56 |
| Non-malignant | 0.51 | 0.50 | 0.86 | 0.13 | 0.78 |
| Glioma | 0.15 | 0.30 | 0.12 | 0.51 | 0.10 |
| Meningioma | 0.92 | 1.00 | 0.99 | 0.91 | 0.77 |
| Cranial nerves | 0.35 | 0.12 | 0.64 | 0.15 | 0.91 |
| Malignant non-glioma of the brain | 0.24 | 0.57 | 0.65 | 0.05 | 0.59 |
| Non-malignant non-glioma of the brain | 0.93 | 0.99 | 0.70 | 0.89 | 0.45 |
| ***B: 20 equal sized categories*** | | | | | |
| Intracranial CNS tumors | 0.83 | 0.01 | 0.89 | 0.70 | 0.24 |
| Malignant | 0.24 | 0.41 | 0.45 | 0.67 | 0.04 |
| Non-malignant | 0.93 | 0.09 | 0.91 | 0.81 | 0.74 |
| Glioma | 0.75 | 0.45 | 0.51 | 0.64 | 0.27 |
| Meningioma | 0.51 | 0.97 | 0.84 | 0.91 | 0.19 |
| Cranial nerves | 0.72 | 0.06 | 0.98 | 0.76 | 0.80 |
| Malignant non-glioma of the brain | 0.52 | 0.99 | 0.25 | 0.40 | 0.16 |
| Non-malignant non-glioma of the brain | 0.80 | 0.84 | 0.42 | 0.46 | 0.85 |

**Supplement table 2**: Linear associations between time-weighted average air pollution over 1, 5 and 10-year periods and risk of intracranial CNS tumors, Denmark 1989-2014

|  |  |  | **1-yr time weighted average exposure** | |  | **5-yr time weighted average exposure** | |  | **10-yr time weighted average exposure** | |
| --- | --- | --- | --- | --- | --- | --- | --- | --- | --- | --- |
| **Air pollutant** | | **IQR (µg/m3)** | **OR pr IQR^a^** | **95% CI** |  | **OR pr IQR^a^** | **95% CI** |  | **OR pr IQR^a^** | **95% CI** |
| **Intracranial CNS tumors** | | | | | | | | | | |
|  | NO_x_ | 18.86 | 1.002 | (0.984-1.020) |  | 1.007 | (0.988-1.026) |  | 1.011 | (0.992-1.030) |
|  | NO_2_ | 10.78 | 1.019 | (0.985-1.054) |  | 1.027 | (0.993-1.062) |  | 1.031 | (0.997-1.066) |
|  | BC | 0.39 | 1.011 | (0.991-1.031) |  | 1.015 | (0.995-1.035) |  | 1.016 | (0.996-1.037) |
|  | PM_2.5_ | 5.39 | 1.017 | (0.956-1.082) |  | 1.000 | (0.937-1.066) |  | 1.010 | (0.944-1.080) |
|  | O_3_ | 9.72 | 0.971 | (0.936-1.006) |  | 0.970 | (0.936-1.005) |  | 0.967 | (0.934-1.002) |
| **Malignant** | |  |  |  |  |  |  |  |  |  |
|  | NO_x_ | 18.86 | 1.012 | (0.985-1.039) |  | 1.017 | (0.989-1.045) |  | 1.026 | (0.998-1.056) |
|  | NO_2_ | 10.78 | 1.026 | (0.977-1.079) |  | 1.031 | (0.981-1.084) |  | 1.042 | (0.992-1.095) |
|  | BC | 0.39 | 1.023 | (0.994-1.053) |  | 1.026 | (0.997-1.056) |  | 1.034 | (1.005-1.065) |
|  | PM_2.5_ | 5.39 | 0.998 | (0.911-1.092) |  | 0.988 | (0.900-1.085) |  | 1.021 | (0.926-1.126) |
|  | O_3_ | 9.72 | 0.973 | (0.924-1.025) |  | 0.972 | (0.923-1.023) |  | 0.962 | (0.913-1.012) |
| **Non-malignant** | | |  |  |  |  |  |  |  |  |
|  | NO_x_ | 18.86 | 0.994 | (0.969-1.019) |  | 0.999 | (0.973-1.026) |  | 0.998 | (0.972-1.025) |
|  | NO_2_ | 10.78 | 1.015 | (0.969-1.063) |  | 1.025 | (0.978-1.073) |  | 1.022 | (0.976-1.070) |
|  | BC | 0.39 | 1.000 | (0.973-1.028) |  | 1.005 | (0.978-1.033) |  | 1.000 | (0.972-1.028) |
|  | PM_2.5_ | 5.39 | 1.036 | (0.951-1.128) |  | 1.010 | (0.924-1.104) |  | 1.000 | (0.912-1.096) |
|  | O_3_ | 9.72 | 0.966 | (0.919-1.016) |  | 0.967 | (0.921-1.016) |  | 0.972 | (0.926-1.021) |
| **Glioma** | |  |  |  |  |  |  |  |  |  |
|  | NO_x_ | 18.86 | 1.008 | (0.977-1.041) |  | 1.010 | (0.977-1.044) |  | 1.017 | (0.983-1.052) |
|  | NO_2_ | 10.78 | 1.016 | (0.959-1.077) |  | 1.018 | (0.960-1.079) |  | 1.026 | (0.969-1.087) |
|  | BC | 0.39 | 1.024 | (0.990-1.059) |  | 1.025 | (0.991-1.060) |  | 1.028 | (0.993-1.063) |
|  | PM_2.5_ | 5.39 | 0.932 | (0.838-1.037) |  | 0.916 | (0.821-1.024) |  | 0.938 | (0.836-1.053) |
|  | O_3_ | 9.72 | 0.982 | (0.925-1.044) |  | 0.981 | (0.924-1.042) |  | 0.973 | (0.916-1.033) |
| **Meningioma** | | | | | | | |  |  |  |
|  | NO_x_ | 18.86 | 1.000 | (0.965-1.036) |  | 1.006 | (0.970-1.044) |  | 1.009 | (0.972-1.047) |
|  | NO_2_ | 10.78 | 1.067 | (1.000-1.139) |  | 1.084 | (1.016-1.157) |  | 1.083 | (1.016-1.154) |
|  | BC | 0.39 | 1.016 | (0.977-1.057) |  | 1.015 | (0.976-1.056) |  | 1.016 | (0.977-1.057) |
|  | PM_2.5_ | 5.39 | 1.129 | (1.000-1.275) |  | 1.101 | (0.971-1.248) |  | 1.088 | (0.955-1.239) |
|  | O_3_ | 9.72 | 0.924 | (0.861-0.992) |  | 0.921 | (0.859-0.987) |  | 0.923 | (0.862-0.988) |
| **Cranial Nerves** | | | | | | | | | | |
|  | NO_x_ | 18.86 | 0.996 | (0.937-1.057) |  | 0.991 | (0.928-1.057) |  | 0.970 | (0.908-1.036) |
|  | NO_2_ | 10.78 | 0.984 | (0.886-1.093) |  | 0.983 | (0.883-1.094) |  | 0.952 | (0.858-1.058) |
|  | BC | 0.39 | 0.989 | (0.926-1.057) |  | 0.969 | (0.904-1.039) |  | 0.943 | (0.879-1.011) |
|  | PM_2.5_ | 5.39 | 1.080 | (0.889-1.311) |  | 1.012 | (0.823-1.245) |  | 0.963 | (0.776-1.194) |
|  | O_3_ | 9.72 | 0.987 | (0.882-1.105) |  | 1.006 | (0.899-1.126) |  | 1.046 | (0.936-1.169) |
| **Malignant Non-glioma tumors of the brain** | | | | |  |  |  |  |  |  |
|  | NO_x_ | 18.86 | 1.017 | (0.966-1.071) |  | 1.035 | (0.982-1.090) |  | 1.049 | (0.996-1.106) |
|  | NO_2_ | 10.78 | 1.039 | (0.942-1.147) |  | 1.065 | (0.965-1.175) |  | 1.084 | (0.983-1.194) |
|  | BC | 0.39 | 1.019 | (0.964-1.077) |  | 1.032 | (0.978-1.089) |  | 1.051 | (0.996-1.110) |
|  | PM_2.5_ | 5.39 | 1.214 | (1.018-1.449) |  | 1.220 | (1.020-1.459) |  | 1.267 | (1.053-1.524) |
|  | O_3_ | 9.72 | 0.975 | (0.878-1.082) |  | 0.955 | (0.861-1.058) |  | 0.937 | (0.846-1.037) |
| **Non-Malignant Non-glioma tumors of the brain** | | | | | | | | | | |
|  | NO_x_ | 18.86 | 0.988 | (0941-1.037) |  | 0.991 | (0.946-1.039) |  | 0.995 | (0.950-1.042) |
|  | NO_2_ | 10.78 | 0.962 | (0.883-1.048) |  | 0.958 | (0.879-1.044) |  | 0.969 | (0.890-1.054) |
|  | BC | 0.39 | 0.985 | (0.965-1.038) |  | 1.006 | (0.961-1.053) |  | 1.003 | (0.956-1.053) |
|  | PM_2.5_ | 5.39 | 0.866 | (0.727-1.031) |  | 0.869 | (0.732-1.033) |  | 0.897 | (0.759-1.060) |
|  | O_3_ | 9.72 | 1.005 | (0.918-1.101) |  | 1.018 | (0.929-1.117) |  | 0.994 | (0.920-1.073) |

a: interquartile range (IQR) and percentiles calculated for the 10-year period among all controls.

Matched on age, sex and month of birth and adjusted for marital status, occupational status, personal income, region of origin and area level information on % of parish population with income in lowest quartile, unemployed, manual labor, retired, basic education, living in social housing, owning their own dwelling, single parent families, previously convicted, of Danish origin.

**Supplement table 3:** Linear associations between mean air pollution (10 years before diagNOsis) and incidence of intracranial CNS tumors with and without adjustment for drug use, Denmark 1996-2014

|  |  |  | **Model 3^a^** | |  | **Model 4^b^** | |
| --- | --- | --- | --- | --- | --- | --- | --- |
| **Air pollutant** | | **IQR (µg/m^3^)** | **OR pr IQR** | **95%CI** |  | **OR pr IQR** | **95%CI** |
| **Intracranial CNS tumors** *(Case: 16579 Controls: 29148)* | | | | | | | |
|  | BC | 0.39 | 1.017 | (0.992-1.042) |  | 1.017 | (0.992-1.042) |
|  | NO_x_ | 18.88 | 1.010 | (0.986-1.034) |  | 1.010 | (0.986-1.034) |
|  | NO_2_ | 10.78 | 1.034 | (0.994-1.075) |  | 1.034 | (0.994-1.075) |
|  | O_3_ | 9.72 | 0.967 | (0.928-1.009) |  | 0.967 | (0.928-1.009) |
|  | PM_2.5_ | 5.40 | 1.013 | (0.931-1.103) |  | 1.013 | (0.931-1.102) |
| **Malignant** *(Case: 7431 Controls: 13087)* | | | | | | | |
|  | BC | 0.39 | 1.042 | (1.005-1.080) |  | 1.042 | (1.005-1.081) |
|  | NO_x_ | 18.88 | 1.032 | (0.996-1.070) |  | 1.033 | (0.997-1.071) |
|  | NO_2_ | 10.78 | 1.054 | (0.993-1.118) |  | 1.055 | (0.995-1.119) |
|  | O_3_ | 9.72 | 0.955 | (0.898-1.016) |  | 0.954 | (0.896-1.015) |
|  | PM_2.5_ | 5.40 | 1.032 | (0.911-1.170) |  | 1.033 | (0.911-1.171) |
| **Non-malignant** *(Case: 9148 Controls: 16061)* | | | | | | | |
|  | BC | 0.39 | 0.994 | (0.960-1.029) |  | 0.992 | (0.958-1.027) |
|  | NO_x_ | 18.88 | 0.992 | (0.960-1.024) |  | 0.990 | (0.959-1.023) |
|  | NO_2_ | 10.78 | 1.017 | (0.965-1.072) |  | 1.015 | (0.963-1.070) |
|  | O_3_ | 9.72 | 0.978 | (0.924-1.035) |  | 0.980 | (0.926-1.038) |
|  | PM_2.5_ | 5.40 | 0.993 | (0.885-1.114) |  | 0.989 | (0.881-1.110) |
| **Glioma** *(Case: 5701 Controls: 9904)* | | | | | | | |
|  | BC | 0.39 | 1.038 | (0.995-1.084) |  | 1.039 | (0.996-1.085) |
|  | NO_x_ | 18.88 | 1.022 | (0.979-1.067) |  | 1.024 | (0.980-1.069) |
|  | NO_2_ | 10.78 | 1.040 | (0.971-1.114) |  | 1.042 | (0.973-1.116) |
|  | O_3_ | 9.72 | 0.964 | (0.897-1.036) |  | 0.962 | (0.895-1.034) |
|  | PM_2.5_ | 5.40 | 0.982 | (0.846-1.140) |  | 0.984 | (0.847-1.142) |
| **Meningioma** *(Case: 4665 Controls: 8261)* | | | | | | | |
|  | BC | 0.39 | 1.032 | (0.984-1.083) |  | 1.028 | (0.980-1.078) |
|  | NO_x_ | 18.88 | 1.024 | (0.979-1.070) |  | 1.020 | (0.975-1.067) |
|  | NO_2_ | 10.78 | 1.107 | (1.029-1.190) |  | 1.101 | (1.023-1-185) |
|  | O_3_ | 9.72 | 0.906 | (0.837-0.980) |  | 0.911 | (0.842-0.986) |
|  | PM_2.5_ | 5.40 | 1.175 | (1.001-1.378) |  | 1.163 | (0.990-1.366) |
| **Cranial Nerves** *(Case: 2025 Controls: 3505)* | | | | | | | |
|  | BC | 0.39 | 0.904 | (0.830-0.985) |  | 0.902 | (0.827-0.982) |
|  | NO_x_ | 18.88 | 0.940 | (0.868-1.018) |  | 0.938 | (0.866-1.017) |
|  | NO_2_ | 10.78 | 0.920 | (0.816-1.037) |  | 0.918 | (0.814-1.036) |
|  | O_3_ | 9.72 | 1.084 | (0.954-1.232) |  | 1.085 | (0.954-1.233) |
|  | PM_2.5_ | 5.40 | 0.96 | (0.739-1.248) |  | 0.964 | (0.741-1.253) |
| **Malignant Non-glioma tumors of brain proper**  *(Case: 1750 Controls: 3200)* | | | | | | | |
|  | BC | 0.39 | 1.043 | (0.975-1.116) |  | 1.043 | (0.975-1.116) |
|  | NO_x_ | 18.88 | 1.051 | (0.984-1.122) |  | 1.051 | (0.984-1.122) |
|  | NO_2_ | 10.78 | 1.081 | (0.963-1.214) |  | 1.082 | (0.964-1.215) |
|  | O_3_ | 9.72 | 0.946 | (0.836-1.070) |  | 0.945 | (0.836-1.069) |
|  | PM_2.5_ | 5.40 | 1.111 | (0.879-1.405) |  | 1.112 | (0.879-1.406) |
| **Non-malignant Non-glioma tumors of the brain proper**  *(Case: 2438 Controls: 4278)* | | | | | | | |
|  | BC | 0.39 | 0.983 | (0.922-1.048) |  | 0.985 | (0.924-1.050) |
|  | NO_x_ | 18.88 | 0.967 | (0.910-1.027) |  | 0.969 | (0.912-1.029) |
|  | NO_2_ | 10.78 | 0.932 | (0.841-1.032) |  | 0.935 | (0.844-1.036) |
|  | O_3_ | 9.72 | 1.048 | (0.939-1.169) |  | 1.044 | (0.936-1.165) |
|  | PM_2.5_ | 5.40 | 0.764 | (0.612-0.954) |  | 0.765 | (0.612-0.955) |

a: Matched on age, sex and month of birth and adjusted for individual marital status, occupational status, personal income, region of origin and area level information on % of parish population with income in lowest quartile, unemployed, manual labor, retired, basic education, living in social housing, owning their own dwelling, single parent families, previously convicted, of Danish origin

b: Model 3 with additional adjustment for forever redeeming 2 prescriptions within a year for Aspirin, Non-aspirin NSAID, HRT, Antidiabetic medication or anti allergic medication.

**Supplement table 4:** Summaries of results from our previous studies rescaled to the IQRs of the present study: PM_2.5_=5.39µg/m^3^, NO_x_=18.86 µg/m^3^, NO_2_=10.78 µg/m^3^.

|  | **End point** |  | **N Cases** |  |  |  |
| --- | --- | --- | --- | --- | --- | --- |
| Raaschou-nielsen et al 2011[1] - *Cohort. Population: Danish Diet Cancer and health cohort. Follow-up period 2000-2009. Covariates: Age and occupation in oil refineries* | | | | | | |
|  | Malignant neoplasm of brain and other parts of nervous system (ICD7: 193) | | 95 | NO_x_ | IRR: | 1.17 (1.04-1.31) |
|  |  |  |  |  |  |  |
| Poulsen et al 2016[2] - *Case Control. Population: Danish population. Follow-up 2000-2009. Covariates: Sex, year of birth, years of living in highly urbanized municipalities.* | | | | | | |
|  | Tumors situated in the brain (ICD10: C71.0-C71.9. D33.0.-D33.2. D43.0-D43.2) | | 4183 | NO_x_ | OR: | 1.02 (0.97-1.07) |
|  | Malignant (ICD10: C71.0-C71.9) | | 3220 | NO_x_ | OR: | 1.02 (0.96-1.08) |
|  | Non-malignant (ICD10: D33.0.-D33.2. D43.0-D43.2) | | 963 | NO_x_ | OR: | 1.03 (0.91-1.15) |
|  | Glioma (ICD-O-3 topography: C71.0–71.9 and morphology: 9380/3–9481/3) | | 2596 | NO_x_ | OR: | 0.97 (0.90-1.04) |
|  | Non-glioma | | 1587 | NO_x_ | OR: | 1.08 (1.00-1.17) |
|  |  | | | | | |
| Andersen et al[3] - *Escape study. pooled European cohort including Danish Diet Cancer and Health cohort. Covariates: age. sex. education. occupation in petrochemical industry. area-level SES* | | | | | | |
|  | Intracranial CNS tumors (ICD10: C70.0. C71.0-C71.9.C72.2-C72.5. D32.0. D33.0-D33.3.D42.0. D43.0-D43.3) | | 466 | PM_2.5_ | HR: | 1.14 (0.59-2.21) |
|  |  |  |  | NO_2_ | HR: | 1.04 (0.82-1.34) |
|  |  |  |  | NO_x_ | HR: | 1.01 (0.85-1.21) |
|  | Malignant (ICD10: C70.0. C71.0-C71.9.C72.2-C72.5) | | 190 | PM_2.5_ | HR: | 0.98 (0.60-1.62) |
|  |  |  |  | NO_2_ | HR: | 1.04 (0.81-1.34) |
|  |  |  |  | NO_x_ | HR: | 1.05 (0.87-1.26) |
|  | Non-malignant (ICD10: D32.0. D33.0-D33.3. D42.0.D43.0-D43.3) | | 176 | PM_2.5_ | HR: | 1.13 (0.43-2.95) |
|  |  |  |  | NO_2_ | HR: | 0.95 (0.70-1.29) |
|  |  |  |  | NO_x_ | HR: | 0.97 (0.76-1.23) |
|  | Situated in brain (ICD10: C71.0-C71.9. D33.0-D33.2. D43.0-D43.2) | | 188 | PM_2.5_ | HR: | 0.87 (0.37-2.03) |
|  |  |  |  | NO_2_ | HR: | 1.24 (0.90-1.70) |
|  |  |  |  | NO_x_ | HR: | 1.11 (0.89-1.39) |
|  | Meningioma (ICD10: C70.0. D32.0.D42.0) | | 115 | PM_2.5_ | HR: | 2.72 (0.86-8.66) |
|  |  |  |  | NO_2_ | HR: | 0.98 (0.60-1.62) |
|  |  |  |  | NO_x_ | HR: | 0.99 (0.67-1.47) |

1. Raaschou-Nielsen O, Andersen ZJ, Hvidberg M, Jensen SS, Ketzel M, Sorensen M, Hansen J, Loft S, Overvad K, Tjonneland A: **Air pollution from traffic and cancer incidence: a Danish cohort study**. *Environ Health* 2011, **10**:67.

2. Poulsen AH, Sorensen M, Andersen ZJ, Ketzel M, Raaschou-Nielsen O: **Air pollution from traffic and risk for brain tumors: a nationwide study in Denmark**. *Cancer Causes Control* 2016, **27**(4):473-480.

3. Andersen ZJ, Pedersen M, Weinmayr G, Stafoggia M, Galassi C, Jorgensen JT, Sommar JN, Forsberg B, Olsson D, Oftedal B *et al*: **Long-term exposure to ambient air pollution and incidence of brain tumor: the European Study of Cohorts for Air Pollution Effects (ESCAPE)**. *Neuro Oncol* 2018, **20**(3):420-432.
